# Significant changes in lower limb alignment due to flexion and rotation—a systematic 3D simulation of radiographic measurements

**DOI:** 10.1007/s00167-022-07302-x

**Published:** 2023-01-03

**Authors:** Josef Brunner, Maximilian Jörgens, Maximilian Weigert, Hannah Kümpel, Nikolaus Degen, Julian Fuermetz

**Affiliations:** 1 Department of Orthopaedics and Trauma Surgery, Musculoskeletal University Center Munich (MUM), University Hospital, LMU, Marchioninistr. 15, 81377 Munich, Germany; 2 Statistical Consulting Unit StaBLab, LMU, Munich, Germany; 3 Department of Trauma Surgery, BG Unfallklinik Murnau, Murnau, Germany

**Keywords:** 3D simulation, Radiographic measurement, Coronal alignment, Lower limb rotation, Knee flexion

## Abstract

**Background:**

Many radiographic lower limb alignment  measurements are dependent on patients’ position, which makes a standardised image acquisition of long-leg radiographs (LLRs) essential for valid measurements. The purpose of this study was to investigate the influence of rotation and flexion of the lower limb on common radiological alignment parameters using three-dimensional (3D) simulation.

**Methods:**

Joint angles and alignment parameters of 3D lower limb bone models (*n* = 60), generated from computed tomography (CT) scans, were assessed and projected into the coronal plane to mimic radiographic imaging. Bone models were subsequently rotated around the longitudinal mechanical axis up to 15° inward/outward and additionally flexed along the femoral intercondylar axis up to 30°. This resulted in 28 combinations of rotation and flexion for each leg. The results were statistically analysed on a descriptive level and using a linear mixed effects model.

**Results:**

A total of 1680 simulations were performed. Mechanical axis deviation (MAD) revealed a medial deviation with increasing internal rotation and a lateral deviation with increasing external rotation. This effect increased significantly (*p* < 0.05) with combined flexion up to 30° flexion (− 25.4 mm to 25.2 mm). With the knee extended, the mean deviation of hip–knee–ankle angle (HKA) was small over all rotational steps but increased toward more varus/valgus when combined with flexion (8.4° to − 8.5°). Rotation alone changed the medial proximal tibial angle (MPTA) and the mechanical lateral distal femoral angle (mLDFA) in opposite directions, and the effects increased significantly (*p* < 0.05) when flexion was present.

**Conclusions:**

Axial rotation and flexion of the 3D lower limb has a huge impact on the projected two-dimensional alignment measurements in the coronal plane. The observed effects were small for isolated rotation or flexion, but became pronounced and clinically relevant when there was a combination of both. This must be considered when evaluating X-ray images. Extension deficits of the knee make LLR prone to error and this calls into question direct postoperative alignment controls.

**Level of evidence:**

III (retrospective cohort study).

**Supplementary Information:**

The online version contains supplementary material available at 10.1007/s00167-022-07302-x.

## Introduction

Despite the availability of three-dimensional (3D) imaging techniques such as magnetic resonance imaging (MRI), computed tomography (CT), EOS^®^ 2D/3D imaging, and digital volume tomography (DVT), preoperative surgical planning is still commonly performed on two-dimensional (2D) long-leg radiographs (LLRs) [5]. The main advantages are standardized, fast and easy image acquisition, as well as broad availability, with standard values for lower limb alignment established over decades [21]. Furthermore, LLRs can identify anatomic variations of the femur and the tibia with high sensitivity by easily assessing the mechanical axis [21]. Additionally, intraoperative fluoroscopic images can be compared with these preoperative images [9, 23].

The standardized observation procedure of LLRs is in upright standing position, with the knee fully extended and a centralised patella in the frontal plane [21]. Many patients with axial deformities, osteoarthritis, total knee arthroplasty (TKA) or other causes of partial immobility cannot fully extend their knees. Yet, 2D X-ray projection images change depending on the patient’s position and are influenced by rotation and flexion [1, 5, 8, 11, 25].

This leads to difficulties in reliably performing LLRs with limited comparability of pre- and postoperative images [2]. The use of LLR is, therefore, questionable for accurate surgical planning in cases of severe deformities or acute injuries, where standardized positioning is not possible [10, 15].

Several studies have investigated the influence of either rotation or flexion on lower limb alignment measurements, and two studies examined combined effects on some of the common radiographic alignment parameters, using a synthetic bone model and 3D simulation programs. Following biomechanical and kinematic considerations, these combined effects were considered to be much greater than those of rotation or flexion alone [10, 13].

Therefore, there was an urgent need to investigate how strong the combined effects were within a larger population and so the focuses of several studies on different clinically important mechanical measures [HKA (hip–knee–ankle angle), MPTA (medial proximal tibial angle), mLDFA (mechanical lateral distal femoral angle), MAD (mechanical axis deviation)] were systematically merged together in a comprehensive manner [11, 13, 16, 23].

To date, there is no study yet, that examined these postulated combined effects due to rotation and flexion on various established mechanical alignment parameters based on virtual CT models. The aim of this study was to quantify the influence of combined rotation and flexion of the lower limb on common alignment parameters using 3D simulation. Based on biomechanical and kinematic considerations, combined effects were assumed to be much greater than from rotation or flexion alone.

## Materials and methods

**Fig. 1:**
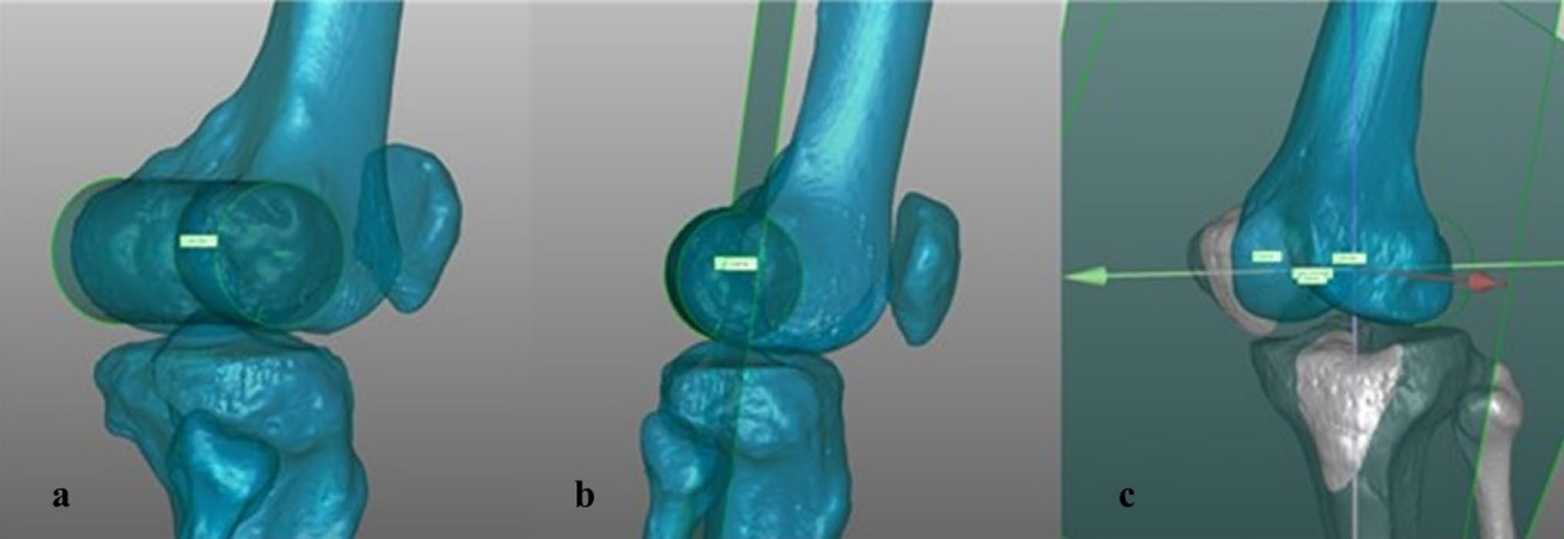
Definition of coordinate system—3D model of the right knee joint; left **a**: implementation of the *x*-axis (medial–lateral), “best fit” cylinder of the femoral epicondyles with the transepicondylar centre vector as best approximation of the knee’s flexion axis [17, 22]; middle **b**: implementation of the *z*-axis (longitudinal): intersecting plane between *x*-axis and the FHC as best approximation of the MFA; right **c**: implementation of the *y*-axis (anterior–posterior): recrossing the *x*- and *z*-axes incorporating the FNP → best approximation of the centre of the knee [5, 18]

For this software and program-based simulation study, 60 3D bone models of the lower limb were used, that were created from existing anonymized CT-data of 30 randomly selected patients (18–50 years) showing alignment parameters within the range of reported norm values and indicating the absence of any severe deformity in coronal neutral position (Table 1) [21]. To cover side differences between left and right limbs, both sides of each of the 30 patients were included. Exclusion criteria were advanced osteoarthritis of the hip joint and knee joint, radiographic evidence of previous realignment surgery, fractures, any lower extremity joint replacement, and age above 50 years. Physiological homogeneity of the selected patient collective was chosen to test the hypothesis, before deformities and more variable coronal alignment could be investigated [7, 19]. Digital 3D copies were processed using the validated rendering software program, Mimics 14.0 (Materialize, Leuven, Belgium), for segmentation and calculation of the CT images and subsequently using the Geomagic Studio 2014 (3D Systems, Morrisville, NC, USA) software to create a 3D geometry of the leg [5]. A standardized new coordinate system was set in every model and enabled us to relate positional changes due to flexion and rotation back to the physiological neutral position (Fig. 1). According to the methods of Miranda et al. the coordinate system was implemented based on mechanical axes, principal mass and cylindrical surface fitting [17]. Considering the need for accurate measurements, a method that uses coordinates established with high accuracy and reliability in previous publications, was chosen [5].

**Table 1:** Summary of alignment measurements of the models for simulation (*n* = 60); *HKA* hip–knee–ankle angle, *MPTA* medial proximal tibial angle, *MAD* mechanical axis deviation, *mLDFA* mechanical lateral distal femoral angle

	HKA (in °)	MPTA (in °)	mLDFA (in °)	MAD (in mm)
Mean	180.1	87.7	87.2	6.2
Minimum	171.3	82.3	83.1	− 11.5
Maximum	187.7	92.7	92.9	28.8
Standard deviation (SD)	± 3.1	± 2.6	± 2.2	± 8.4

### Definition of angles and points

As it was aimed to quantify changes in angular measurements, validated and publication-based 3D landmarks were integrated into all models [5]. Their projection into the coronal plane finds an approximate equivalent to the 2D landmarks of D. Paley, which are commonly used in this research field [5, 21]. A python code was written in which the y-coordinate was set to zero and all measurements were automatically projected into the coronal plane to mimic radiographic imaging [5, 16, 23].To evaluate the changes in alignment, after every simulation step, angles and distances were automatically evaluated with another python script, and measured results were statistically analysed. By convention, negative measurements indicated the lower extremity to be internally rotated and positive measurements externally rotated around the longitudinal mechanical axis [8].

The centre of the femoral head (FHC), the femoral notch point (FNP) and the centre of the tibial articular surface of the ankle joint (AJC) were chosen to define the mechanical axis (MA) [8, 21]. According to the study by Moreland et al., the current study utilized the FNP as the femoral centre of the knee and the centre between the tibial spines on the tibial surface as the tibial centre of the knee (TKC) [5, 18]. To measure the HKA, the connecting lines between FHC and FNP, as well as between TKC and AJC were created. This angle is defined as the medial angle between those two vectors [10, 21]. MAD was calculated as the distance of the MA from the centre of the knee joint. The most distal points of the femoral condyles and the most proximal lateral and medial points of the tibia were necessary to describe mLDFA and MPTA [5, 8, 21].

### Simulation of flexion and rotation

**Fig. 2:**
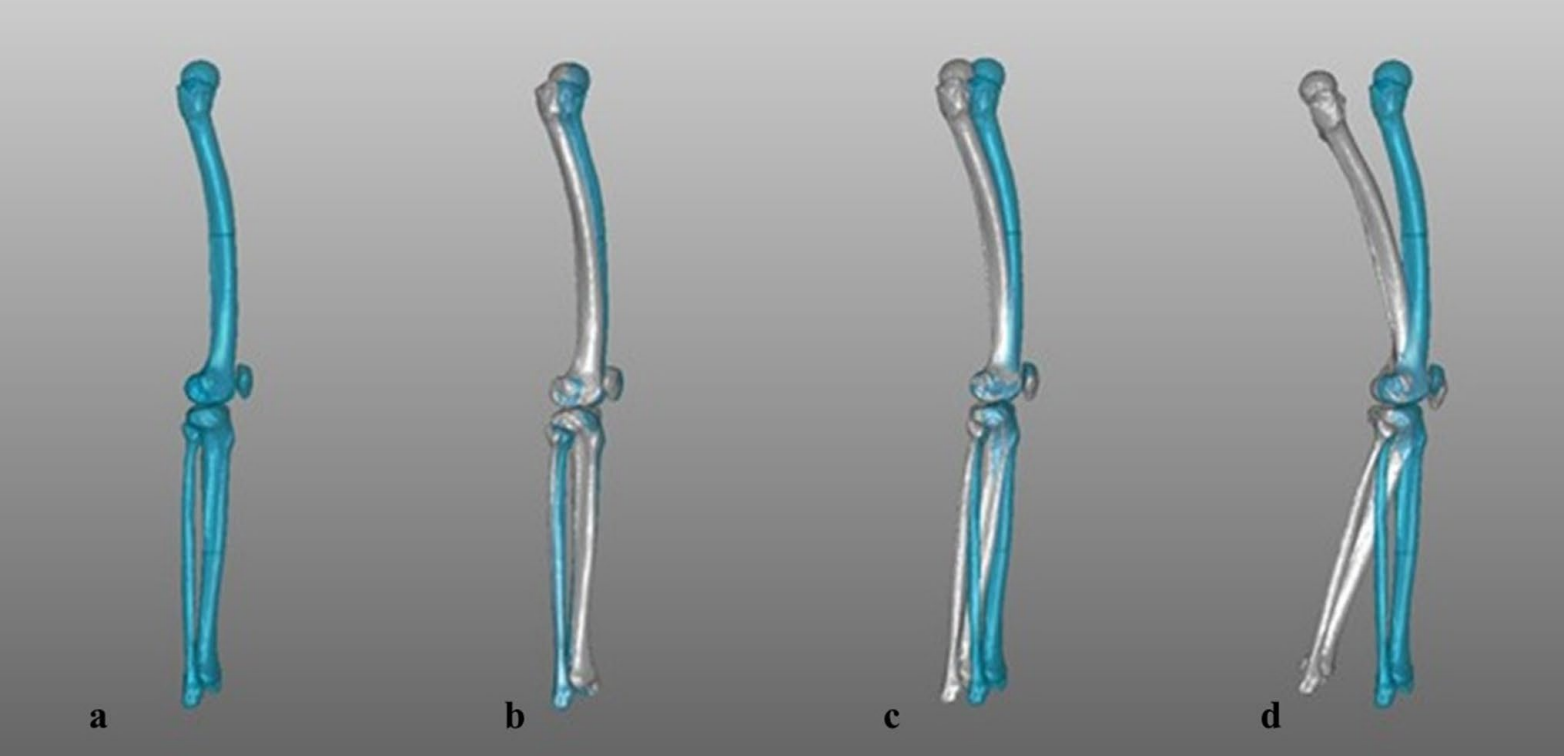
Right bone model in different positions, lateral view (blue: reference zero position; white: flexed/rotated model); **a** zero position; **b** 15° external rotation; **c** 10° flexion; **d** 30° flexion with 15° internal rotation

The models were then aligned to the new coordinate system and a neutral origin position (0° flexion, 0° rotation) was set. Next, all models were rotated around the longitudinal MA in 5° increments up to 15° internally and 15° externally and additionally flexed in 10° steps along the femoral transepicondylar axis up to 30° (Fig. 2). For every model, 28 combinations of flexion and rotation were simulated, which led to 1680 positions in total.

Half of the flexion was performed on the femoral part of the model and half on the tibial part in reverse direction. The division of motion and the determination of the vertical long axis from the FHC to the ankle joint were performed according to the methods of Jud et al. to obtain a realistic position compared with radiographs [10]. Furthermore, the screw home motion of the knee joint in the last 20° of extension by additionally internally rotating the tibia 5° during flexion was simulated [24].

### Statistical analysis

The impact of different degrees of flexion and rotation on the measured clinical parameters MAD, HKA, mLDFA, and MPTA was analysed on a descriptive and a model-based level. Descriptive analyses focused on mean differences to the values observed without any flexion or rotation. Additionally, an individual linear mixed effects model was fitted for each of the clinical parameters (MAD, HKA, mLDFA and MPTA) using the R package lme4 [3]. With measurements given in increments of 5 and 10 degrees, respectively, rotation and flexion were treated as categorical variables with reference categories R0 and F0 for modelling purposes. In addition, a fixed effect for the leg side and a random intercept on patient level (*n* = 30) were included in the model. Likelihood ratio tests were applied to test for the estimated rotation and flexion effects as well as their interaction. The significance level was set to *α* = 0.05 for all conducted hypothesis tests. To account for multiple testing, all *p* values were adjusted via the Benjamini–Hochberg method [4]. Marginal effects in terms of predicted values were visualized using the R package sjPlot [14].

All results and the related statistical calculations can also be found in the appendix, supplemental file area.

## Results

**Fig. 3:**
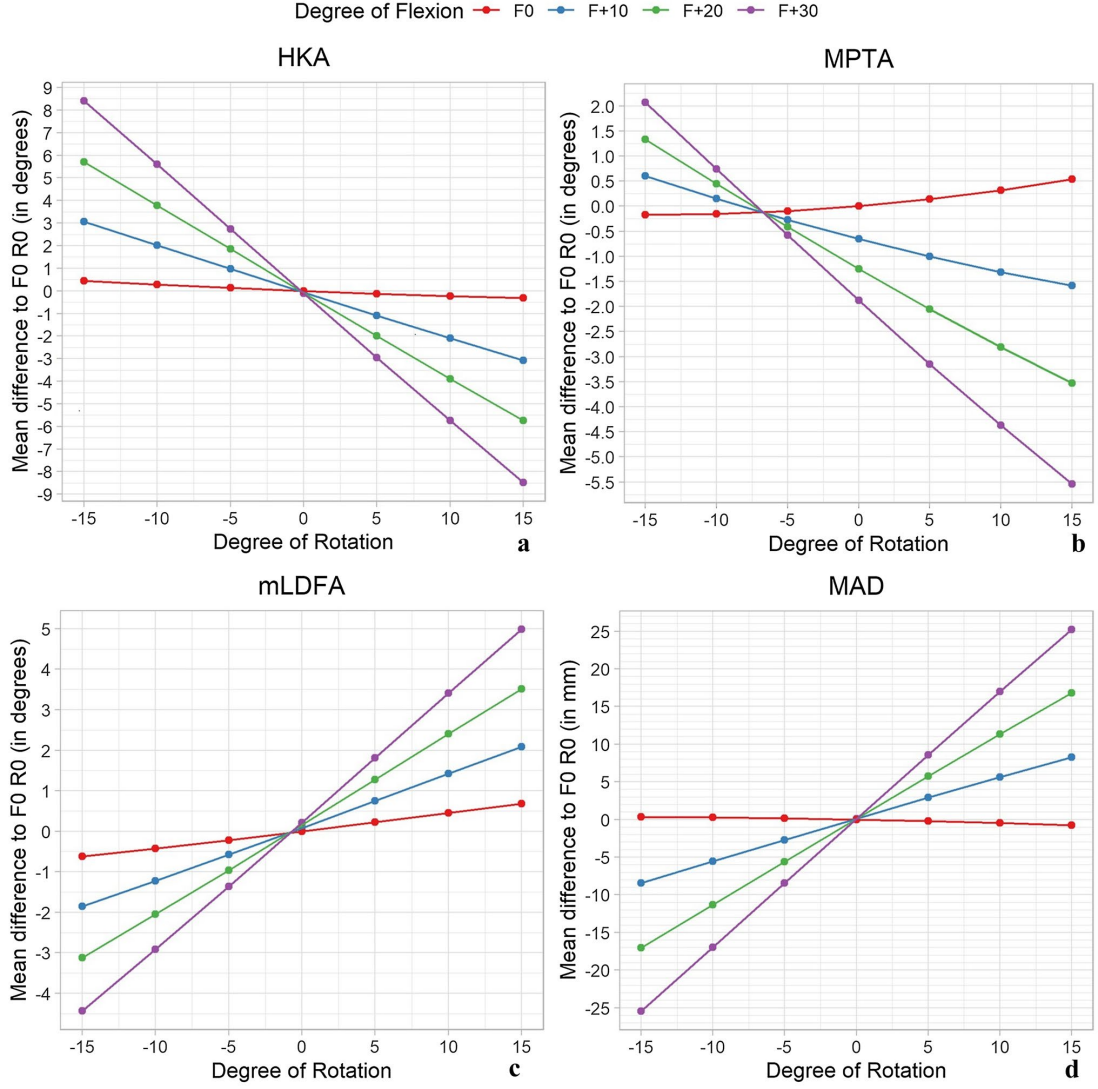
Mean differences to the zero-position dependent on rotation and flexion effects measured in the simulation for HKA angle (**a**), MPTA (**b**), mLDFA (**c**) and MAD (**d**); Negative values caused by internal rotation and positive values by external rotation. Coloured graphs represent different states of flexion; *x*-axis different states of rotation; *MPTA* medial proximal tibial angle, *HKA* hip–knee–ankle angle, *MAD* mechanical axis deviation, *mLDFA* mechanical lateral distal femoral angle

**Fig. 4:**
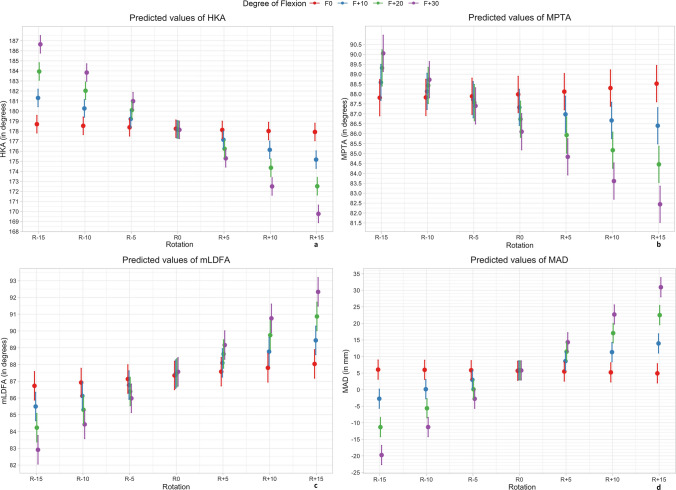
Predicted values (with CI 95%) of the HKA angle (**a**), MPTA (**b**), mLDFA (**c**) and MAD (**d**); Rotation and flexion effects based on linear mixed model calculation; Negative rotation values  representing internal rotation, positive external rotation; *MPTA* medial proximal tibial angle, *HKA* hip–knee–ankle angle, *MAD* mechanical axis deviation, *mLDFA* mechanical lateral distal femoral angle

All examined parameters showed highly remarkable deviations, comparing values for zero position and positions with flexion and rotation of the bone models (Figs. 3 and 4). No significant effect was found for most parameters with either rotation or flexion alone, but a significantly increasing effect in combination (*p* < 0.05). Estimated plots of the deviation to zero position for every examined combination of rotation and flexion are shown in the appendix (Figs. 5, 6, 7, 8).

Consistently high values for the conditional determination coefficient *R*^2^, which describes the proportion of the variance explained by the combination of fixed effects (rotation and flexion) and random effects (patient effects) indicated that the established linear regression model was a very good approximation to the actual measured values from the simulation [20].

In the zero-position mean value for HKA angle was 180.1° (SD: ± 3.1°). The linear regression model (*R*^2^ conditional = 0.93) calculated approximately a 0.03° change of measured HKA per degree limb rotation with extended knee. When the knee was 30° flexed, the change per degree increased up to 0.6° (Fig. 4).

The MPTA with measured mean zero position of 87.7° (SD: ± 2.6°) showed different tendencies to alter during simulation. With the knee extended, the MPTA decreased with internal rotation and increased with external rotation (Figs. 3 and 4). The linear regression model (*R*^2^ conditional = 0.85) calculated a 0.02° change of MPTA per degree limb rotation. Additionally, the MPTA angle was most affected by flexion alone, compared with all other angles. A flexion of 10 degrees led to a decrease of the angle by 0.7° and 30° flexion by 1.9°. Interestingly, external rotation in combination with flexion had a higher impact on the differences to the neutral position than with internal rotation.

For the mLDFA a mean zero position of 87.2° (SD: ± 2.2°) was measured. With internal rotation the angle decreased and increased with external rotation. The linear regression model (*R*^2^ conditional = 0.89) calculated a 0.04° change of measured mLDFA per degree limb rotation when the knee was extended. For flexion 30° the linear regression model calculated a change of 0.3° per degree additional limb rotation (Fig. 3).

The mean MAD was measured at 6.2 mm (SD: ± 8.4 mm), which is in the range Paley et al. reported as a physiological norm value 8 mm ± 7 mm [21]. Among all studied angles, the MAD was the parameter with the highest difference between singular effects and combined effects. As it is shown in Fig. 3, the combination of both led to estimated variations of approximately 25 mm in each direction.

## Discussion

The most important finding of the study, was the confirmation, that rotation or flexion alone have little effect on limb alignment parameters, but when combined, these effects can reach clinically relevant values very quickly. The demonstrated results provide a useful tool for clinicians to estimate the change in lower limb alignment parameters when radiographs are affected by extension deficits or malrotation.

In the following, our findings were compared to several studies that investigated similar questions regarding the effect of rotation on limb alignment and used comparable methods.

Lonner et al. used a singular sawbone model of a well-aligned TKA and quantified the effect of lower limb rotation and 10° flexion on the anatomic alignment [13]. They saw significant changes in tibial alignment through the additional effect of flexion, with an overall total variation in HKA of 8° from 20° internal to 20° external rotation (*p* < 0.05). In line with these findings, our values for HKA changed within a comparable range.

Similar, Kannan et al. solely investigated the influence of external rotation with additional flexion on the HKA. They addressed a similar question and concluded that flexion and rotation alone influenced the HKA < 1°, but a combination of both altered it substantially [11].

Jud et al. questioned if constitutional varus or valgus alignment (± 9°) influences the effect of flexion and rotation on alignment parameter relevantly. After performing rotation and flexion on virtual 3D models in incremental steps up to 30°, there were no relevant interpatient differences in changes of the HKA [10]. In contrast to this study, limb alignment parameters and joint angles of most of the patients investigated in our study were within the standard range (Table 1). Thus, it can be concluded that our results are probably applicable to more severe deformities.

Following Radtke et al. and several other studies, 5° steps of incremental rotation up to 15° maximum were chosen to obtain comparable results [11, 13, 23]. The linear regression model calculated a 0.05° change of the MPTA per 1° limb rotation [23]. The trend towards varus/valgus by rotation was the same in our results, but the effect was slightly smaller with 0.02° change per 1° of rotation angle in full extension.

Jamali et al. predicted a significant effect on all parameters except mLDFA and anatomic lateral distal femoral angle (aLDFA) [8]. In contrast to this, no significant changes by rotation of only 3° in full extension were found. Different flexion angles seemed to be mainly responsible for the different results in the neutral origin position compared to the study of Jamali et al. thus amplifying the effects of rotation.

Compared to other simulation methods, such as sawbone models, cadaveric or in vivo studies, there are some limitations regarding an appropriate biomechanical simulation. Normally LLRs are taken in weight bearing upright position with the patella pointing forward, whereas the used CT data were acquisitioned in supine position [6, 26]. Anyway, CT imaging uses a linear radiation source, whereas the X-ray beam is divergent. Therefore, CT images do not exhibit typical distortions compared to X-ray images. However, newer imaging methods such as EOS or DVT also use linear radiation [25].

Complex underlying bone deformities may significantly alter measurements, wherefore our findings are only valid for patients without severe deformities in the coronal plane. The degree of final external rotation of the tibia was set to 5° to postulate a screw home motion [12].

With these results, underlying flexion and rotation effects for patients without severe deformities can be approximated and values for calculating alignment parameters in neutral position are provided. As LLRs can only estimate rotation based on patella position or fibula overlap, while information on flexion is missing, EOS or DVT can provide coronal LLR along with sagittal and axial information that will allow the demonstrated results to be implemented in future studies and clinical practice. In addition, this study underlines the relevance of 3D imaging and 3D preoperative planning, especially when standardised positioning for LLR is not possible.

## Conclusion

Axial rotation and flexion of the 3D lower limb have a huge impact on the projected 2D alignment measurements in the coronal plane. The observed effects were small for isolated rotation or flexion, but became pronounced and clinically relevant when there was a combination of both. This must be considered when evaluating X-ray images. Extension deficits of the knee make LLR prone to error and this calls into question direct postoperative alignment controls.

## Supplementary Information

Below is the link to the electronic supplementary material.

Supplementary file1 (DOCX 1216 KB)

## Data Availability

All data generated or analysed during this study are included in this published article (and its supplementary information files).

## Abbreviations

AJC: Ankle joint centre
CT: Computed tomography
DVT: Digital volume tomography
FHC: Femoral head centre
FNP: Femoral notch point
HKA: Hip–knee–ankle angle
aLDFA: Anatomic lateral distal femoral angle
LPFA: Lateral proximal femoral angle
LLR: Long-leg radiograph
wbLLR: Weight-bearing long-leg radiograph
MA: Mechanical axis
MAD: Mechanical axis deviation
MFA: Mechanical femoral axis
MTA: Mechanical tibial axis
ML: Medial lateral
mLDFA: Mechanical lateral distal femoral angle
MPFA: Medial proximal femoral angle
MPTA: Medial proximal tibial angle
MRI: Magnetic resonance imaging
NSA: Neck shaft angle
TKA: Total knee arthroplasty
TKC: Tibial knee centre
2D: Two-dimensional
3D: Three-dimensional
