# Supplementary material for: Significant changes in lower limb alignment due to flexion and rotation—a systematic 3D simulation of radiographic measurements
Source: Knee Surg Sports Traumatol Arthrosc. 2023 Jan 3;31(4):1483–90. doi: 10.1007/s00167-022-07302-x (PMC10050026; doi:10.1007/s00167-022-07302-x)
Supplement: Supplementary file 1 — Supplementary file1 (DOCX 1216 KB) [file 167_2022_7302_MOESM1_ESM.docx]

# **Appendix**

## **Supplementary material**

Raw data and R-Code-File are available on: http://dx.doi.org/10.17632/sy5sxn5svj.1

The following figures 5-8 show estimated values for the changes of the HKA, MPTA, mLDFA and MAD angle for single and combined effects of flexion and rotation.

All effects are shown as deviations due to flexion and rotation in comparison to the zero position with full extension and without rotation. Estimating combined effects calculated by the linear regression model, every single effect shown in the plot must be summarized.

For example, the combined effect of 10° internal rotation [R-10] and 20° flexion [F+20] is calculated in the following manner: [R-10] + [F+20] + [R-10 * F+20]

Additionally, for right legs the side factor [Seite] must be considered and added as well.

These instructions are valid for all the following estimated plots.

Different levels of significance are indicated by stars. If there is further interest into datasets and codes from this publication, there is a Mendeley Data repository which can be found by following the mentioned link above.


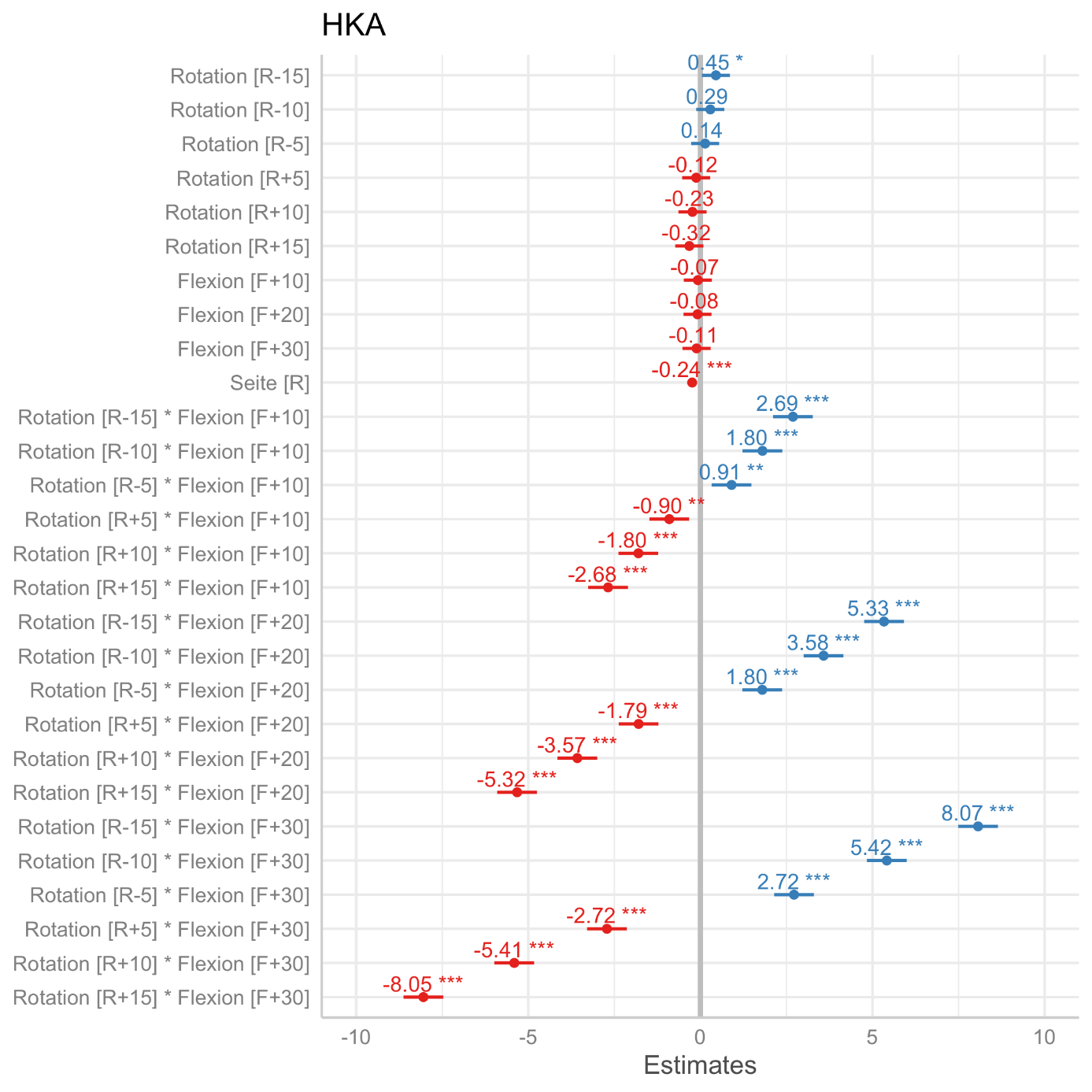


**Figure 5: Estimated values of the effects of rotation and flexion on the HKA angle;**

(***≙ p-value [0; 0,001]; ** ≙ p-value [0,001; 0,01]; * ≙ p-value [0,01; 0,05])


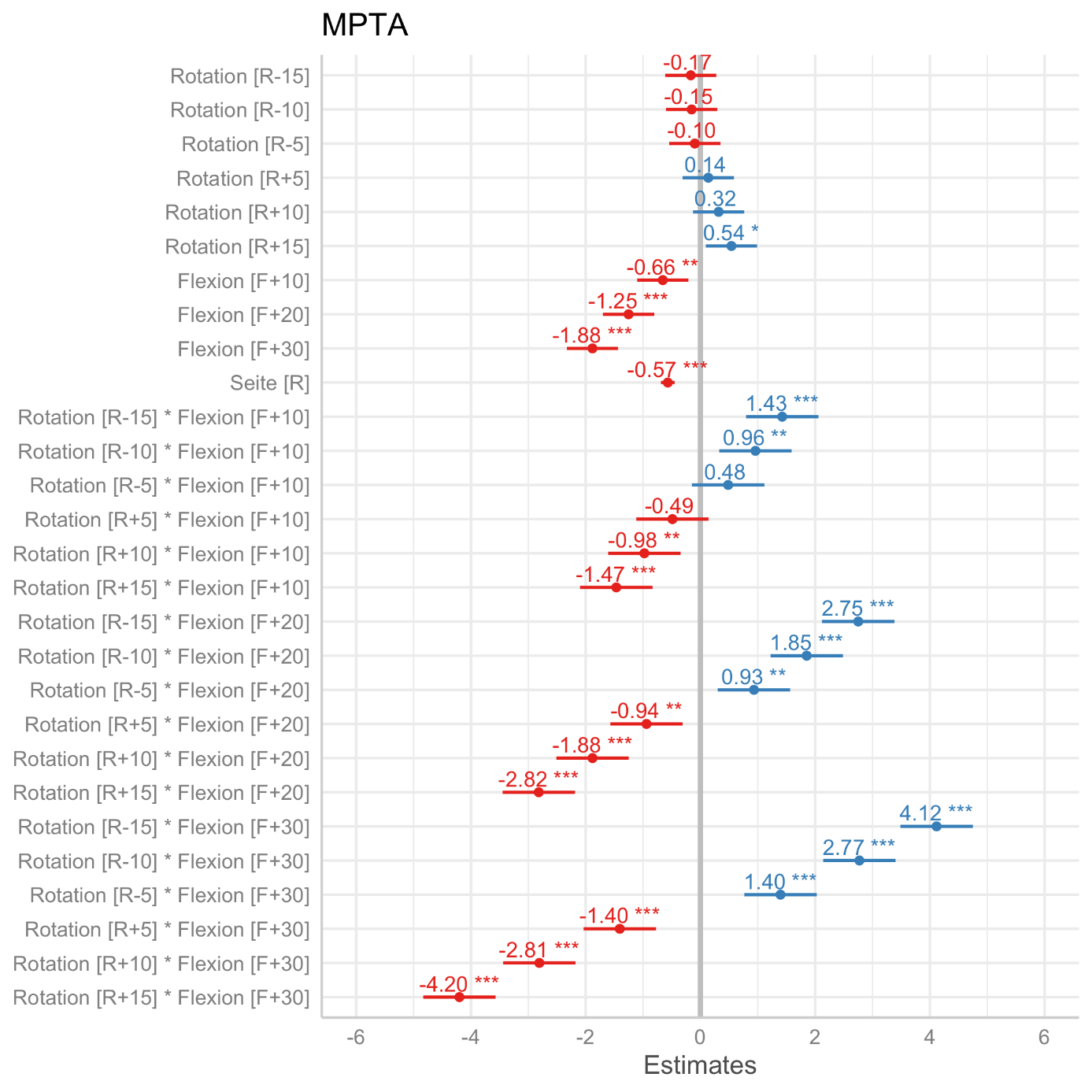


**Figure 6: Estimated values of the effects of rotation and flexion on the MPTA angle;** (***≙ p-value [0; 0,001]; ** ≙ p-value [0,001; 0,01]; * ≙ p-value [0,01; 0,05])


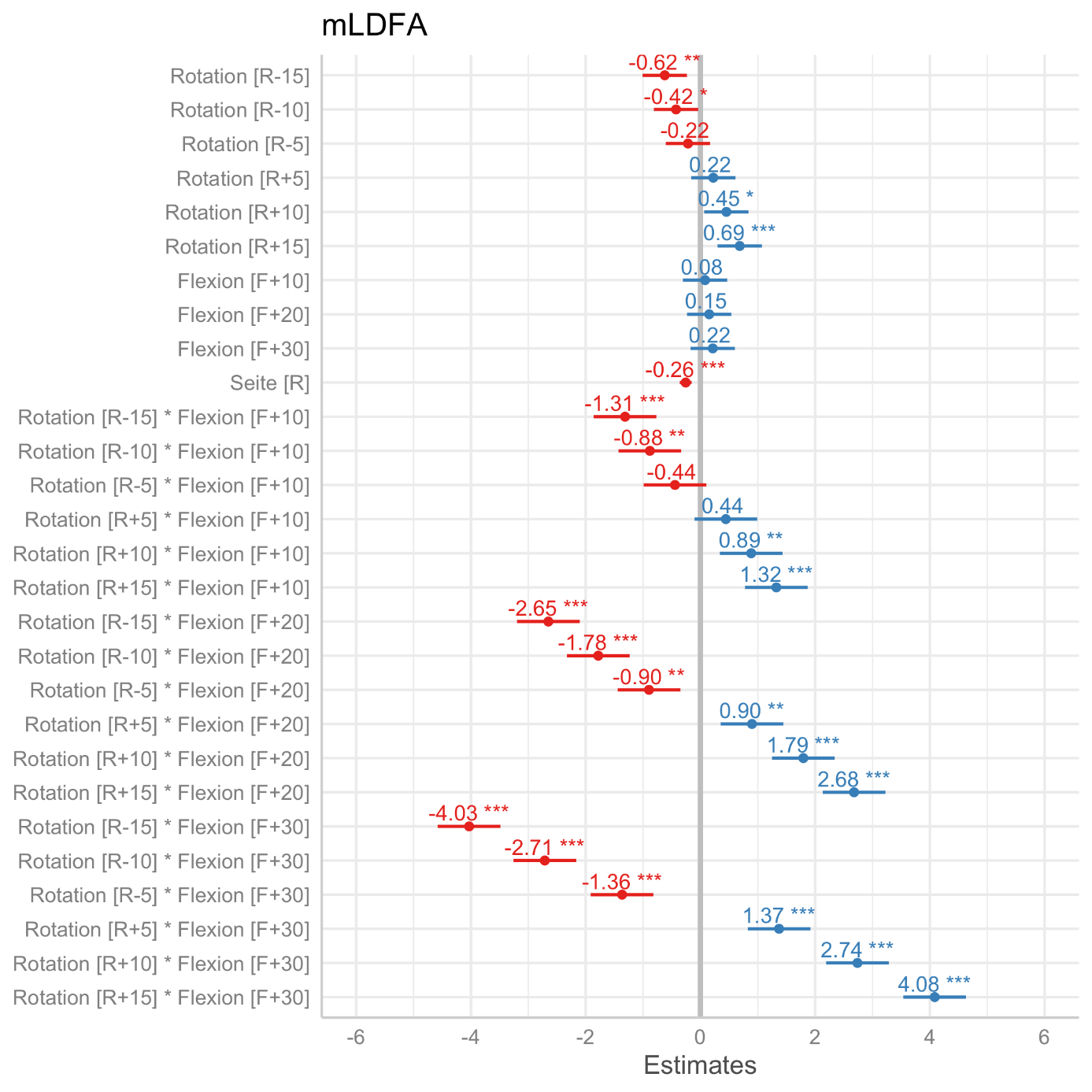


**Figure 7: Estimated values of the effects of rotation and flexion on the mLDFA angle;**

(*****≙** p-value [0; 0,001]; ****** ≙ p-value [0,001; 0,01]; ***** ≙ p-value [0,01; 0,05])


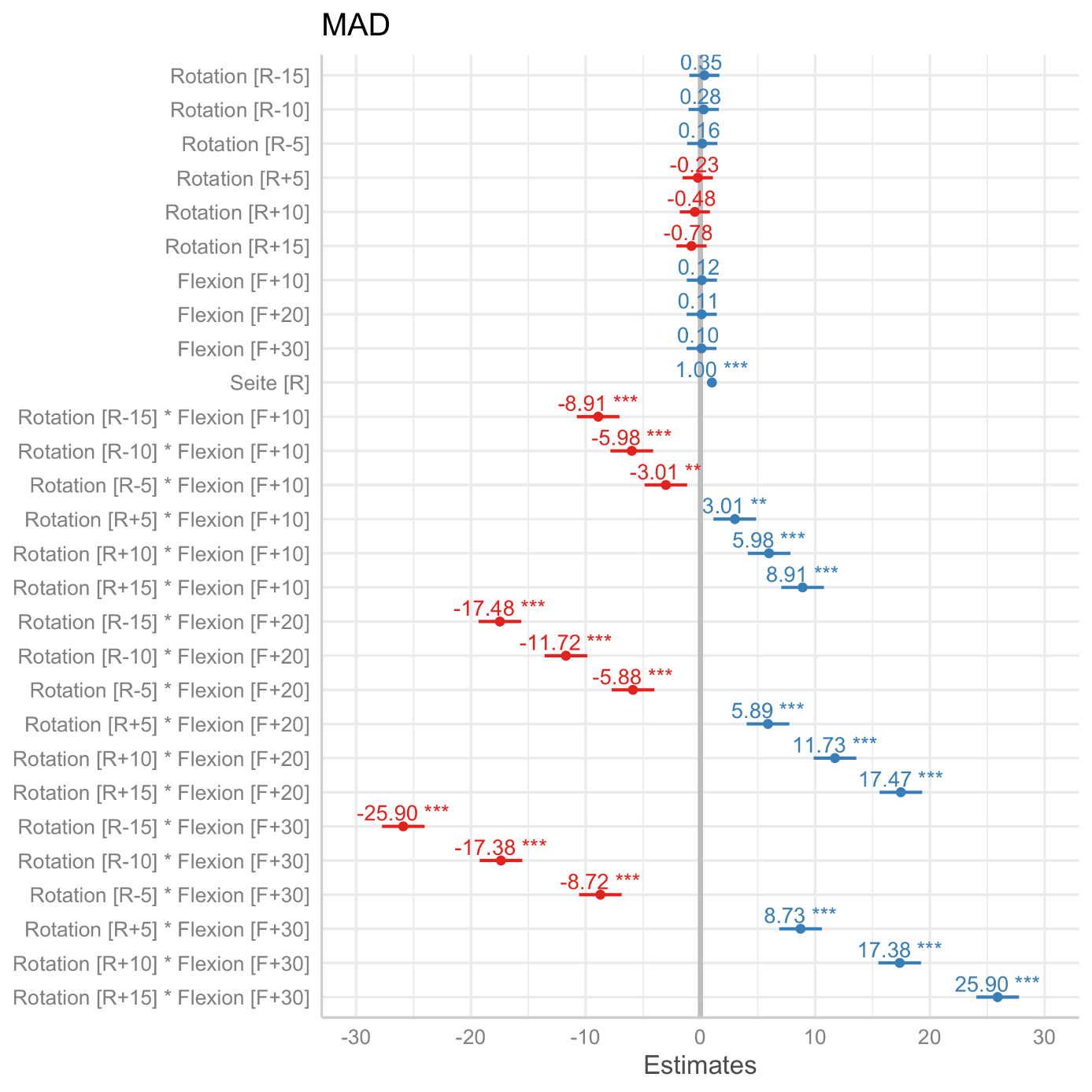


**Figure 8: Estimated values of the effects of rotation and flexion on the MAD;** (***≙ p-value [0; 0,001]; ** ≙ p-value [0,001; 0,01]; * ≙ p-value [0,01; 0,05])
